# Asthma and Multimorbidity Amongst Ethnic Minority Groups in High Income Countries

**DOI:** 10.1111/cea.70329

**Published:** 2026-05-15

**Authors:** Mamidipudi Thirumala Krishna, Saibal Moitra, Anna Freeman, Ben Ainsworth, Rebecca Knibb, Ramesh Kurukulaaratchy, Adel H. Mansur

**Affiliations:** ^1^ Department of Immunology and Immunotherapy, School of Infection, Inflammation and Immunology, College of Medicine and Health University of Birmingham Birmingham UK; ^2^ Department of Allergy and Immunology University Hospitals Birmingham NHS Foundation Trust Birmingham UK; ^3^ Division of Allergy and Immunology, Department of Respiratory Medicine, Apollo Multispeciality Hospitals Kolkata West Bengal India; ^4^ School of Clinical and Experimental Sciences, Faculty of Medicine University of Southampton Southampton UK; ^5^ National Institute for Health and Care Research (NIHR) Southampton Biomedical Research Centre at University Hospital Southampton NHS Foundation Trust Southampton UK; ^6^ School of Psychology, Faculty of Environmental and Life Sciences University of Southampton Southampton UK; ^7^ Institute of Health and Neurodevelopment Aston University Birmingham UK; ^8^ The David Hide Asthma & Allergy Research Centre, St Mary's Hospital Newport UK; ^9^ Birmingham Regional Severe Asthma Service, Birmingham Heartlands Hospital, University Hospitals Birmingham NHS Foundation Trust and Institute of Inflammation and Ageing, School of Medical Sciences University of Birmingham Birmingham UK

**Keywords:** afro‐Caribbean, allergy, Asthma, black, clinical outcome, deprivation, disparities, ethnicity, multimorbidity, south Asian

## Abstract

Asthma is one of the commonest noncommunicable diseases worldwide. Poor clinical outcomes have been reported in asthma amongst ethnic minority groups (EMGs) and reasons are likely to be multifactorial. There is a suggestion that underlying disease may behave differently amongst EMGs, alongside other factors including deprivation, cultural, religious, social, literacy, patient beliefs, healthcare access, treatment adherence, alongside greater burden of multimorbidity. Multimorbidity (presence of ≥ 2 long‐term health conditions) in asthma is increasingly known to contribute to greater asthma burden, with differences in multimorbidity burden and patterns seen across ethnicities with a tight association with deprivation. Earlier onset of multimorbidity with relatively reduced survival has been reported amongst EMG patients, particularly those from a deprived background compared to White patients. Data regarding asthma, multimorbidity and ethnicity are scant, but emerging data suggest that multimorbidity is heterogenous, and that ethnic background is associated with variations in asthma outcomes and multimorbidity phenotypes. What is currently lacking is population‐based research with a joined‐up interrogation of primary care and secondary care databases to determine the prevalence and patterns of T2 and non T2 multimorbidity in asthma amongst EMGs, and analysis of patterns of associations and link to socio‐demographic variables and clinical outcomes. Deeper insight into views of EMG patients and their carers regarding lived experiences of asthma and multimorbidity management, as well as those of healthcare professionals is needed, to allow development of culturally tailored resources. These studies are likely to shape a holistic culturally tailored multidimensional and an equitable approach to management of asthma with multimorbidity amongst EMGs.

## Key Messages

- Social deprivation, ethnic minority groups (EMGs), and multimorbidity associate with poorer clinical outcomes in asthma.
- Epidemiological research is needed to investigate the prevalence and patterns of multimorbidity in asthma in EMGs.
- There is a need for multidimensional culturally tailored approaches for management of asthma in EMGs.

## Graphical abstract

There is a tight intersection between asthma, deprivation, ethnicity, multimorbidity and poor clinical outcomes. An integrated, holistic and culturally tailored approach is needed to improve clinical outcomes amongst ethnic minority groups with asthma and multimorbidity.

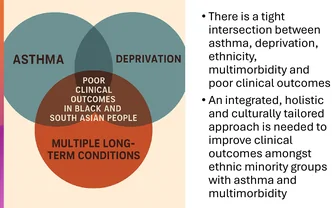

## 1 Ethnicity‐Based Disparities in Asthma in High Income Countries

Asthma is a chronic inflammatory disorder affecting lower airways and is characterised by mucosal inflammation and bronchial hyperreactivity. It is one of the most common noncommunicable diseases affecting over 350 million people worldwide [1]. A previous report in 2018 estimated 65,000 asthma‐related admissions in England annually with a positive correlation (*r* = 0.67) with index of multiple deprivation [2]. Recent data (2022–23) from the UK asthma hospitalisation dashboard reported 92,209 hospitalisations (125.5/100,000) [3].

There is heterogeneity in the nomenclature used to describe ethnicity in High Income Countries. The UK Office of National Statistics 2021 census lists four main categories of ethnicity including Asian (Indian, Pakistani, Bangladeshi and Chinese), Black (African, Caribbean and other Black), White and Mixed or other ethnic groups. In this article ethnic minority groups (EMGs) refers to South Asian and Afro‐Caribbean/Black population. People living in deprived areas are more likely to suffer from acute asthma, and a high proportion of people from EMGs are socioeconomically deprived [4, 5, 6]. The English indices of deprivation 2019 data suggest that 19.3% of Bangladeshi, 31.1% Pakistani and 15.2% of Black people live in 10% of most deprived neighbourhoods [7]. Approximately 13% (8.17 m) of the UK population are from EMGs, and 1 in 5 people in major cities such as London and Birmingham are of South Asian origin.

In a longitudinal cohort study involving UK primary care database, Subramanian et al. reported that the prevalence of allergic rhinitis and asthma amongst the British White population was 11.5% and 7.2% respectively [8]. The prevalence in British South Asian, British Afro‐Caribbean and British Mixed Ethnic groups was 7%, 6% and 5.6% for allergic rhinitis and 7.5%, 7% and 5.7% for asthma respectively [8]. This study showed that the incident risk of allergic rhinitis, asthma, atopic dermatitis and ten organ‐specific and systemic autoimmune conditions were significantly greater amongst British South Asian and British Afro Caribbean people compared to British White people [8]. In the Bradford born cohort involving children of White (*N* = 495), Pakistani (*N* = 961) and other ethnicities (*N* = 190), the prevalence of doctor ‘ever diagnosed asthma’ was 8.9%, 13.4% and 3.2% respectively [9]. In a systematic review and meta‐analysis of 13 prevalence studies (published from 1981 to 2002) in British children, Netuveli et al. reported ethnicity‐based variations [10]. The pooled prevalence (95% CI) of clinician‐diagnosed asthma was 10.6% (4.6%–16.7%), 15.0% (3.5%–26.5%), 7.6% (3.7%–11.4%) in White, Black and South Asians respectively, and the risk (odds ratio 2‐fold higher) of asthma admission was higher amongst Black and South Asian children compared to White ethnicity [10]. The prevalence of asthma is significantly higher amongst Black and Puerto Rican population in USA and similar observations have been reported from Australia [11, 12].

Ethnicity‐based disparities have attracted great attention in High Income Countries including the UK, USA and Australia in view of poor clinical outcomes in asthma, allergic rhinitis, and food allergy [13, 14, 15]. Poor clinical outcomes have been linked to multiple factors including cultural, social, religious, lower literacy, socioeconomic status, deprived area of residence and co‐existing multiple long‐term conditions [6, 13]. Most evidence comes from retrospective observational studies involving large database searches interrogating data from primary and secondary care and few involving prospective surveys, both in paediatric and adult populations.

Ethnicity‐based disparities in aero‐allergen sensitisation have been reported amongst EMGs in High Income Countries. For example, the risk of cockroach sensitisation is 16‐fold higher in African American children and has been linked to rural area of residence (4‐fold higher) and deprivation (12‐fold higher) [16]. In a study involving difficult‐to‐control asthma in central England, Mansur et al. showed that the risk of house dust mite sensitisation is significantly greater amongst British EMGs, most of whom were from South Asian ethnicity [17]. Higher titres of serum specific IgE to dust mite and higher levels of blood eosinophils were detected compared to Caucasian White patients [17].

A number of poor clinical outcomes have been reported amongst people with asthma from EMGs in the UK, USA and Australia [6, 13, 15]. EMG patients have poorer asthma control as evidenced by more frequent visits to the emergency room, greater oral corticosteroid usage, intensive care admissions and higher rates of fatal asthma [13, 15, 18]. Multiple factors may impact these outcomes, including poor treatment adherence and/or engagement with health services and access, more social deprivation associated with EMGs which causes poor quality of life, and/or whether the disease is inherently more severe and complex amongst EMGs [19]. In a recent study in a specialist UK National Health Service Allergy service involving a relatively small cohort of British South Asian patients with food allergy, Osborne et al. reported significantly more frequent allergic reactions alongside poorer asthma and eczema control compared to the Caucasian White population [20]. Furthermore, a higher standardised incident risk of anaphylaxis has been reported amongst British and Australian EMG patients [12, 21]. It is well recognised that both severe anaphylaxis and fatal anaphylaxis are closely associated with poor asthma control [22, 23, 24]. A disproportionately high rate of fatal asthma and fatal anaphylaxis have been reported amongst Black patients and Puerto Rican patients in the USA [25, 26, 27, 28].

The main aim of this narrative review is to examine the published evidence relating to (a) intersectionality of asthma, multimorbidity and ethnicity, (b) the adverse impact of multimorbidity on clinical outcomes in asthma, (c) emerging evidence regarding the benefit of addressing common co‐existing long‐term conditions on clinical outcomes and (d) identifying research gaps relating to asthma and multimorbidity amongst EMGs.

## 2 Contextualising Asthma and Multimorbidity

**FIGURE 1:**
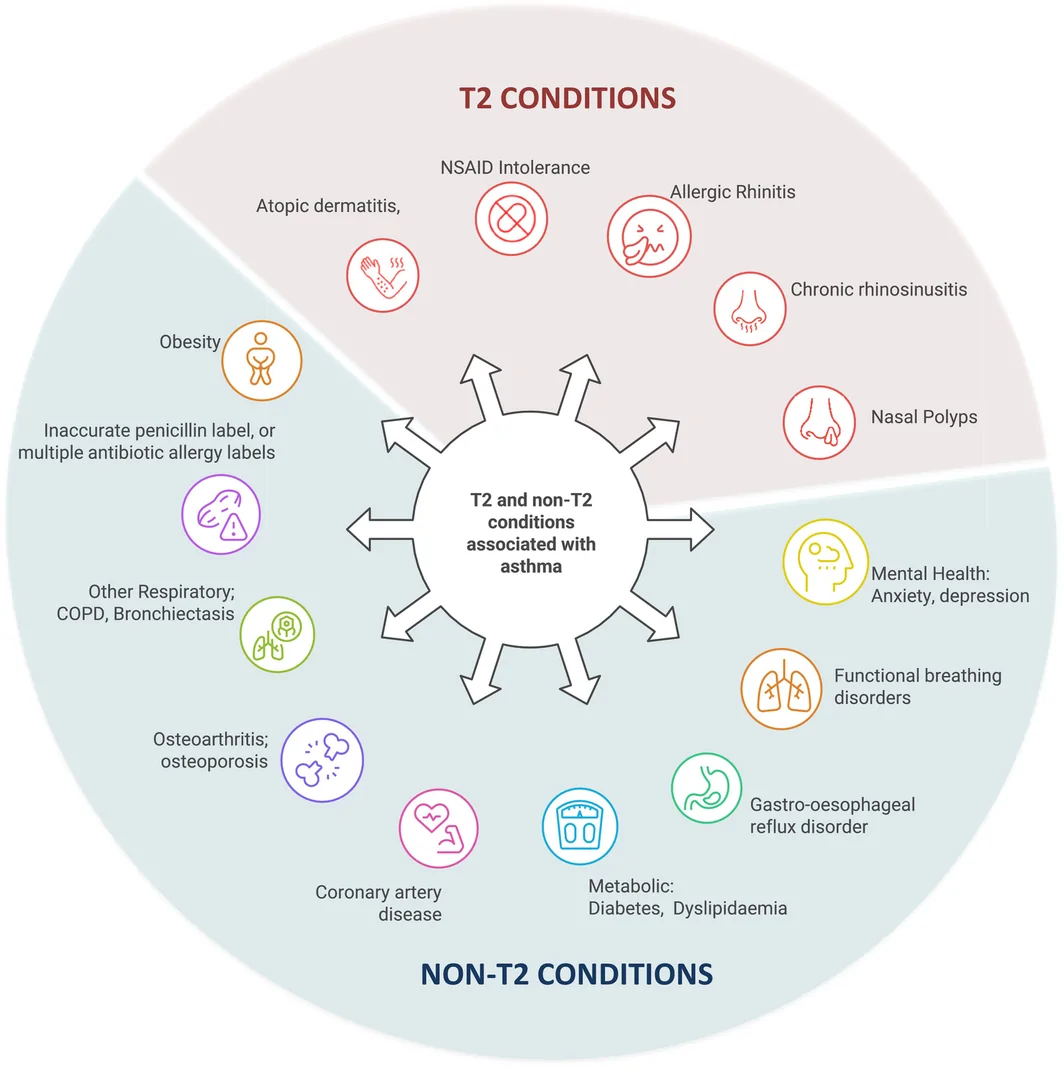
Asthma and associated co‐existing long‐term conditions. Illustration of interplay between asthma and multimorbidity (T2 and non T2). CAD, Coronary artery disease; COPD, Chronic obstructive pulmonary disease; NSAID, Nonsteroidal anti‐inflammatory drugs.

Multimorbidity is defined as the presence of two or more long‐term conditions including physical and mental illnesses in the same patient at a given time [29]. Broadly, there are two types of long‐term co‐existing conditions in asthma (Figure 1), T1 (non‐allergic) and T2 (allergic), and these are discussed in the later sections.

The National Institute of Clinical and Care Excellence published guidelines on clinical assessment and management of multimorbidity given its adverse impact on health‐related quality of life, excess mortality, polypharmacy, enhanced risk of drug‐related adverse events, lesser compliance and higher unplanned use of clinical services, all contributing to excess healthcare costs [29]. Health services in secondary and tertiary care, and clinical guidelines relating to long‐term conditions including asthma are currently single condition‐focused resulting in patients receiving care from multiple specialists, and patients are at a potential risk of fragmented and disjointed care [29, 30]. For example, in the UK National Health Service, a patient with uncontrolled asthma, allergic rhinitis, food allergy, diabetes, obesity and hyperlipidaemia might be attending a specialist allergy, specialist asthma, specialist diabetes and specialist lipid/metabolic clinics, in addition to their primary care reviews.

There are studies which show multimorbidity is more prevalent amongst men, though other studies show the prevalence to be more in women, amongst people from EMGs, those from deprived areas, smokers, obese patients and those with mental health problems [31, 32, 33, 34, 35, 36]. Data from the Global Burden of Disease Study, suggested that cancer, cardiovascular diseases, chronic respiratory diseases including asthma and chronic obstructive pulmonary disease and diabetes were the most common noncommunicable disease conditions linked to death in 2015 in England and in the global adult population [37, 38]. One study in 2020 reported that the overall prevalence of multimorbidity defined as ≥ 2 long‐term conditions was ~15% in England, and the prevalence rate was significantly influenced by age [31]. Asthma, autism and epilepsy dominated in children aged ≤ 19 years, depression and asthma in those 20–49 years, hypertension and depression for those between 50 and 59 years and cardiometabolic conditions and osteoarthritis in those ≥ 60 years [31]. It has also been reported that multimorbidity occurs in 35% and 55% of people between 55–64 years and 65–74 years respectively. Deprivation is one of the most important risk factors for multimorbidity, with a nearly 50% higher risk [39, 40, 41]. In a population‐based study involving England population between 2004 and 2015, the prevalence of basic multimorbidity (≥ 2 chronic conditions) and complex multimorbidity (≥ 3 or more conditions involving 3 body systems) increased from 30.8% and 15.1% to 52.8% and 32.7% respectively [42]. Deprivation was associated with increased incidence of complex multimorbidity with a median seven‐year younger age of onset [42]. Multimorbidity is therefore highly relevant in patients from EMGs, as a large majority are from a lower socio‐economic stratum living in deprived areas, have lesser literacy and this is further compounded by barriers to access elective healthcare and poor compliance with clinical management [4, 33, 43].

A population‐based study interrogating primary and secondary healthcare records in England from 2010 to 2020 involving ~2.5 M people with ≥ 2 long‐term conditions showed that early onset (< 40 years) multimorbidity was more common amongst South Asian (56%) and Black people (59%) compared to Caucasian White people (42%), and that South Asian and Black people died early with a lesser median survival of 9 and 13 years respectively, compared to Caucasian White people [44]. These observations highlight an urgent need for a more nuanced and novel targeted approach to the management of multimorbidity amongst EMGs, specifically focused on prevention, early diagnosis and provision of holistic care, preferably in accessible community settings.

Recent studies have highlighted the importance of recognising the adverse impact of multimorbidity in both children and adults with asthma [6, 45, 46, 47, 48, 49, 50, 51, 52, 53, 54]. T2‐related conditions include allergic rhinitis, atopic dermatitis, food allergy, nasal polyps, and chronic rhinosinusitis and often co‐exist in a significant proportion of patients alongside a number of non‐T2 conditions such as functional breathing disorders, mental illness, obesity, obstructive sleep apnoea, metabolic syndromes including diabetes and dyslipidaemia, cardiovascular disorders, chronic respiratory diseases such as bronchiectasis and chronic obstructive pulmonary disease, chronic pain conditions, osteoporosis, gastro‐oesophageal reflux, and autoimmune conditions (Figure 1). Whilst T2 co‐existing long‐term conditions might be predicted to be part of an atopic diathesis syndrome, the high prevalence of non‐T2 conditions shown by these studies demonstrates that a far wider spectrum of multimorbidity is clinically relevant in such patients. Some of these associations may plausibly reflect shared biological mechanisms not yet delineated or, alternatively, iatrogenic effects such as corticosteroid‐related disease impacts. These studies have shown that numerous individual co‐existing T2 and non‐T2 long‐term conditions as well as cumulative multimorbidity count are significant risk factors for poor asthma outcomes both amongst children and adults including worse exacerbations, lung function, asthma control, and oral corticosteroid dependency [45]. Of note, numerous co‐existing T2 and non‐T2 long‐term conditions were also identified as independent barriers to achieving severe asthma remission with biologic therapies in a recent systematic review and meta‐analysis [55].

The nature of multimorbidity in difficult‐to‐treat/severe asthma and how that is associated with patient outcomes is not well understood. Recently Kurukulaaratchy et al. [46] used data from the UK‐based Wessex AsThma CoHort of difficult asthma (WATCH) to model the association of multimorbidity with asthma severity‐risk in difficult‐to‐treat asthma. Following an iterative approach, they developed a Multimorbidity in Difficult Asthma Score (MiDAS) comprising the seven comorbidities in WATCH that retained significant association with a composite asthma severity measure. Those were rhinitis, gastro‐oesophageal reflux disease, breathing pattern disorder, obesity, bronchiectasis, non‐steroidal anti‐inflammatory drug‐exacerbated respiratory disease, and obstructive sleep apnoea. MiDAS was then tested amongst four international cohorts (USA, Singapore, and 2 in Australia) where it replicated well. Higher MiDAS correlated with poor clinical outcomes including worse quality of life, asthma control, psychological distress and biomarkers of inflammation including leptin, IL‐4, IL‐5 and CRP [46]. Both the WATCH, Australian and USA cohorts included in this study were overwhelmingly White Caucasian, though the US cohort included a substantial minority of Black Americans. Of note, the Singapore cohort showed the weakest replication of MiDAS and had no White patients but was predominantly Chinese or Malay. This emphasises a need to recognise that the nature of multimorbidity in asthma may vary for different ethnic groups. Nevertheless, MiDAS provides a tool for clinicians to start to recognise the presence and potential impact of multimorbidity in their patients with difficult‐to‐treat asthma. It could then act as a prompt to guide more holistic treatment options.

As well as asthma and allergic conditions, there is a high risk of diabetes, metabolic disorders including obesity, hypertension, coronary artery disease, mental health and autoimmune conditions such as rheumatoid arthritis and lupus, amongst EMGs in High Income Countries [8, 56, 57, 58, 59, 60, 61].

Gibson‐Scipio et al. [62] investigated the impact of psychological distress and asthma‐related anxiety with asthma outcomes amongst urban African American young adults with poorly controlled asthma. Applying a multivariable model, they reported that higher somatisation scores and asthma‐related anxiety scores correlated negatively with asthma control and higher asthma attacks. Pate et al. analysed USA National Health Survey interview data 2021 involving 7343 children (age 2–17 years) and adults (≥ 18 years) and analysed the prevalence of ‘asthma only’ and those with asthma and allergy co‐existing long‐term conditions, as per symptoms involving skin, nose and eyes as a surrogate for hay fever, allergic rhinitis, food allergy and eczema [63]. Eight percent of the whole cohort had asthma, and over 50% reported T2 long‐term conditions [63]. The prevalence of asthma–T2 long‐term conditions was significantly higher amongst deprived patients as per federal poverty levels (< 100% vs. ≥ 200%) and non‐Hispanic Black (vs non‐Hispanic White) people [63]. Baptist et al. [5] investigated risk factors for asthma exacerbations amongst American patients with severe asthma in the CHRONICLE cohort (followed from 2018 to 2022). 180 Black and 570 non‐Black (Latino or non‐Hispanic) patients who were not receiving biologics were included in a propensity score analysis involving 7 domains—demographics, spirometry, air pollution exposure, co‐existing long‐term conditions (anxiety, atopic dermatitis or eczema, chronic obstructive pulmonary disease, depression, diabetes, gastroesophageal reflux disease, valvular heart disease, hypertension, and allergic rhinitis), smoking status, socioeconomic status and concurrent maintenance systemic corticosteroid use. This study showed that socioeconomic status was the strongest discriminator of race (C statistic of 0.75), followed by environment (0.65), demographics (0.64), smoking status (0.55), and co‐existing long‐term conditions (0.55) [5].

## 3 Global Evidence Linking Asthma with Multimorbidity and Treatable Traits

The burden, patterns and adverse impact of multimorbidity in asthma and associated treatable traits have attracted much interest globally. In an observational study (2022–2024) involving the Singapore severe asthma registry, Tay et al. [48] reported ethnicity‐based disparities amongst 254 patients. As well as poor asthma clinical outcomes amongst Indian and Malay patients in comparison with Chinese patients, Indian and Malay patients had higher rates of high BMI and diabetes, whilst osteoporosis was significantly more common in Chinese patients.

Bisquera et al. [64] conducted a retrospective cohort study (2005–2020) in an adult British population (*n* = 826,936) in the inner London borough in a primary care setting with a median follow up of 4.2 years. They included 32 long‐term conditions including asthma based on a consensus approach. 631,760 (76%) entered the study with no long‐term condition, 121,424 (15%) with 1 long‐term condition, 41,720 (5%) with 2 long‐term conditions, and 31,966 (4%) with three or more long‐term conditions. At the end of the study period, 24% developed ≥ 1, 5% had resolved long‐term condition, and 3% died. Deprivation, female sex, and Black ethnicity were independently associated with progress from single to two long‐term conditions and shorter period in a healthy state. However, asthma and multimorbidity was specifically not investigated in this study.

Engelkes et al. [65] reported all‐cause mortality in patients (*N* = 586,436; severe asthma—7.3%) with asthma interrogating five European electronic databases from the Netherlands, Italy, UK, Denmark and Spain during the (2008–2013). Age and sex standardised all‐cause mortality rates were 5.2–9.5/1000 person‐years (PY) in asthma, and 11.3–14.8/1000 person‐years in severe asthma and all‐cause mortality rate in the first week following a severe asthma attack was 14.1–59.9/1000 person—years [65]. The main risk factors identified in this study were higher age, male gender, co‐existing long‐term conditions, smoking, and previous severe asthma attacks [65]. Co‐existing long‐term conditions significantly contributing were somewhat variable between countries. For example, in the UK cohort cancer, cerebrovascular disease, cardiovascular disease, obesity, diabetes and smoking status were significantly associated, although cardiovascular disease, obesity and smoking status were not significant in the Danish cohort [65]. Whilst this study provided important insights into asthma and multimorbidity, they were mainly related to the White population.

Pattanaik et al. [66] analysed data from the Study on Global Ageing and Adult Health Wave‐2, involving 11,818 native Indian participants (*N* = 7118; age ≥ 50 years) for prevalence and patterns of multimorbidity relating to co‐occurrence of 8 long‐term conditions including arthritis, stroke, coronary artery disease, diabetes, chronic lung disease, asthma, depression, and hypertension. They found prevalence to be 45% with hypertension (22%) and arthritis (19%) as leading causes, with the former as the most frequent first onset condition. A number of disease dyads were detected with asthma pairing with hypertension.

Park et al. investigated multimorbidity in Korean adults (*N* = 8370; ≥ 50 years) who participated in the Korean National Health and Nutrition Examination Survey (2013–2015) [67]. Ten chronic diseases were included in the study including hypertension, dyslipidaemia, stroke, osteoarthritis, tuberculosis, asthma, allergic rhinitis, depression, diabetes mellitus, and thyroid [67]. Latent class analysis revealed three clusters including ‘relatively healthy group’ (60%), ‘arthritis, asthma, allergic rhinitis, depression and thyroid disease’ group (11.8%) and the ‘cardiometabolic conditions group’ (27.8%). Deprivation was associated with the latter two groups with the ‘arthritis, asthma, allergic rhinitis, depression and thyroid disease’ group significantly associated with poorer quality of life [67].

In a recent UK study published as a conference abstract, Gassase et al. [68] investigated the association between the index of multiple deprivation and clinical outcomes in asthma including exacerbations, primary care consultations, emergency room visits, hospital and intensive care admissions and mortality amongst 787,621 patients and showed that deprivation was significantly associated with increased rates of healthcare utilisation in primary and secondary care, as well as significantly higher risk of asthma‐specific and all‐cause mortality.

Janevic et al. [69] reported challenges faced by 25 African American women with asthma and multimorbidity (mean of 5.7 non‐T2 conditions), with at least one of three non‐T2 conditions including diabetes, heart disease or arthritis in a qualitative study. This study reported an adverse impact of asthma on non‐T2 conditions, as well as the impact of non‐T2 conditions on asthma. The main observations reported were that < 50% considered asthma as their main health problem (‘back seat’ to other conditions). Non‐T2 conditions such as pain‐related conditions and depression adversely impacted cognitive ability and motivation to self‐manage asthma [69]. Limitation of mobility due to chronic conditions and/or polypharmacy impacted directly or indirectly on asthma management [69]. Similarly, acute asthma attacks impacted on the management of other conditions, physical activity and were sometimes deemed to trigger other conditions [69].

In a systematic review including 6 observational studies in High Income countries in children 0–18 years, risk of intensive care admissions for asthma was associated with high salbutamol use, previous hospital admission, deprivation, allergies, Black ethnicity, pneumonia, rural residence, and multimorbidity [27]. The main co‐existing long‐term conditions were prematurity, congenital heart disease and chronic respiratory disorders [27]. Specifically, the authors identified a four‐fold higher risk and seven‐fold higher risk respectively of intensive care admission and mortality amongst Black children compared to White children and highlighted limitations in published evidence in terms of risk of bias and imprecision, and the need for further research to investigate risk factors contributing to intensive care admissions and fatal asthma in the paediatric age group.

Whilst multimorbidity is challenging in Caucasian White patients with asthma, there are additional layers of complexities in patients from EMGs with respect to confounding variables related to deprivation, literacy, access to healthcare, confidence in health services, poor adherence with treatment, language barrier, and the need for a culturally targeted and tailored approach. Current single condition targeted service model puts patients at an enhanced risk of fragmented care, thus contributing to poor treatment adherence and engagement with clinical services, compounding healthcare inequities as patients are required to navigate complex and disjointed healthcare services. It could be argued that a single multidimensional asthma and multimorbidity clinic is likely to offer better continuity of care, trust, confidence, and better engagement with services and improve clinical outcomes.

Treatable traits were defined as potentially modifiable factors which are (a) clinically relevant, (b) identifiable and measurable and (c) treatable. They were broadly categorised as pulmonary, extrapulmonary or behavioural/lifestyle. The treatable traits paradigm in airways disease was first proposed a decade ago as a framework for personalised patient care [70]. Strategies to address treatable traits would necessarily include multimodal approaches to deliver patient advice and support via a multidisciplinary team to manage common co‐existing T2 and non‐T2 conditions including sinonasal disease, gastro‐oesophageal reflux disease, breathing pattern disorder, weight management, osteoporosis, anxiety and depression as well as psychological/behavioural approaches to address contributory lifestyle risk factors such as non‐adherence, smoking or allergen exposure (which, in turn, benefit pulmonary and extrapulmonary factors). Subsequent studies have demonstrated the clinical efficacy of multimodal approaches to address treatable traits in difficult‐to‐treat/severe asthma. In a 16‐week randomised control trial of a nurse‐coordinated multimodal treatment process for Australian patients with difficult‐to‐treat asthma, McDonald et al. demonstrated significant improvements in asthma control and quality of life compared to standard care [71]. This study involved severe asthma patients with a mean ± standard deviation of about 10 ± 3 treatable traits per patient with a reasonable balance between pulmonary and extra pulmonary (4.85 ± 1.86) and behavioural (2.58 ± 1.31) traits. A retrospective analysis of a multidisciplinary approach to treatable traits in difficult‐to‐treat asthma by Lin et al. identified 5 distinct trait profiles (2 airway centric and 3 non‐airway centric) based on assessment for 12 treatable traits [72]. This study was conducted from 2014 to 2022 in a tertiary centre in Melbourne, Australia. It showed that 6 months of trait‐based tailored management was associated with significant improvement in asthma control, quality of life, lung function, exacerbations and oral corticosteroid use. A further study in the same centre by Denton et al. demonstrated that a systematic assessment of severe asthma patients and subsequent targeted management facilitated a 50% reduction in maintenance oral corticosteroid dose (independent of biologic co‐administration), alongside improved asthma symptom control and quality of life as well as exacerbation reduction in over 50% patients [73]. Furthermore, tailored self‐management programmes targeting extrapulmonary symptoms such as anxiety/depression have demonstrated potential to improve asthma quality of life as feasible and pragmatic complementary approaches to medical management [74]. These studies however, involved mainly White patients, and there is an urgent need for such studies amongst EMGs.

Lessons can also be learnt from a French Pharmacoepidemiological nested case control study (Asthma E3N) [51]. This study was largely based in the Caucasian White French population, involving analysis of medications in a large cohort of 17,458 elderly women of whom 4328 had asthma [51]. Three multi‐medication profiles were delineated including few multimorbidity related medications, predominantly allergy related medications and predominantly metabolic multimorbidity related medications, with the latter two groups associated with poor asthma outcomes based on level of asthma therapy, asthma control, asthma exacerbations and asthma quality of life questionnaire [51].

These studies highlight a place for a multidimensional or an integrated holistic management of asthma with multimorbidity amongst EMGs employing a culturally tailored and targeted approach embedding education, primary prevention, secondary prevention, targeting treatable traits and offering basic management of common co‐existing T2 and non‐T2 long‐term conditions. Culturally tailored supportive interventions should address both surface or observable characteristics and deeper structures such as cultural, social, behavioural and environmental characteristics to facilitate better understanding and engagement with clinical services [75]. The main facilitators of self‐management reported in previous studies include culturally and socially adaptable education at a patient level [76]. Community‐based culturally tailored education has been successful in diabetes and cardiovascular disease [77, 78]. Lessons can be drawn from these studies to develop similar strategies for EMG patients with asthma and multimorbidity.

In a cluster randomised control trial involving primary care practices in England and Scotland, Salisbury et al. [79] investigated the impact of 3D (dimensions of health, depression and drugs) interventional care on health‐related quality of life in patients with multimorbidity (≥ 3 long‐term conditions; *N* = 1546; 33 GP practices) and randomised practices to standard of care and 3D model with a 15 month follow up. The vast majority of participants in both groups were Caucasian White with ~3% EMGs or unknown ethnicity. Amongst 11 long‐term health conditions, the majority were cardiovascular or chronic kidney disease in both groups (> 93%–94%), followed by diabetes (52%–54%) and chronic obstructive pulmonary disease or asthma (49%–51%). No differences were detected between the two groups, although 3D care improved patient perceptions of quality of care and there was no specific additional resource utilisation. The authors made a case that provision of patient‐centred care that improves patient experience is one of three basic elements of healthcare [80].

Indeed, qualitative research exploring patient experiences in clinics across 7 European countries found that patients highlighted the need for co‐ordinated, multidisciplinary care that was personalised to their broad range of extra‐pulmonary symptoms [81]. A patient‐centred multimorbidity clinical service has been described in the Danish healthcare system, which involves an interface between primary care and specialist services [82]. This service includes a general physician, pharmacist and occupational therapist reviewing the patient in a single visit taking a holistic approach, formulating a detailed summary followed by a remote discussion with relevant specialists and feedback to patient and family physician [82]. Clinical outcomes and cost‐effectiveness were, however, not reported by the authors.

**FIGURE 2:**
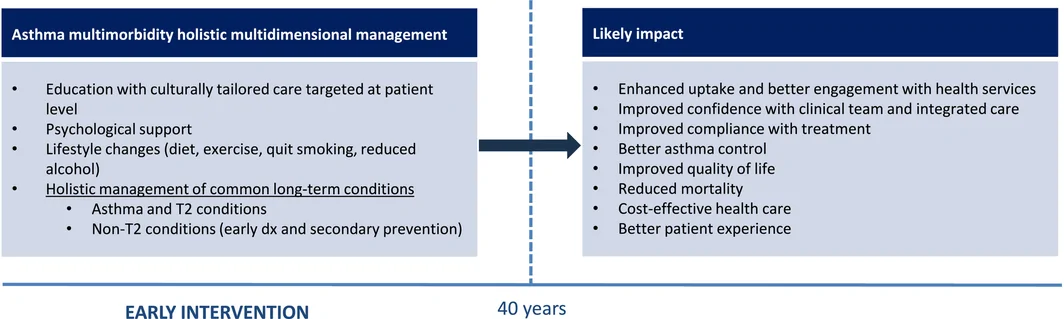
Asthma and multimorbidity holistic multidimensional management and likely impact. Illustration of holistic clinical management of common T2 and non T2 multimorbidity in asthma and potential impact of early interventions on clinical outcomes.

**FIGURE 3:**
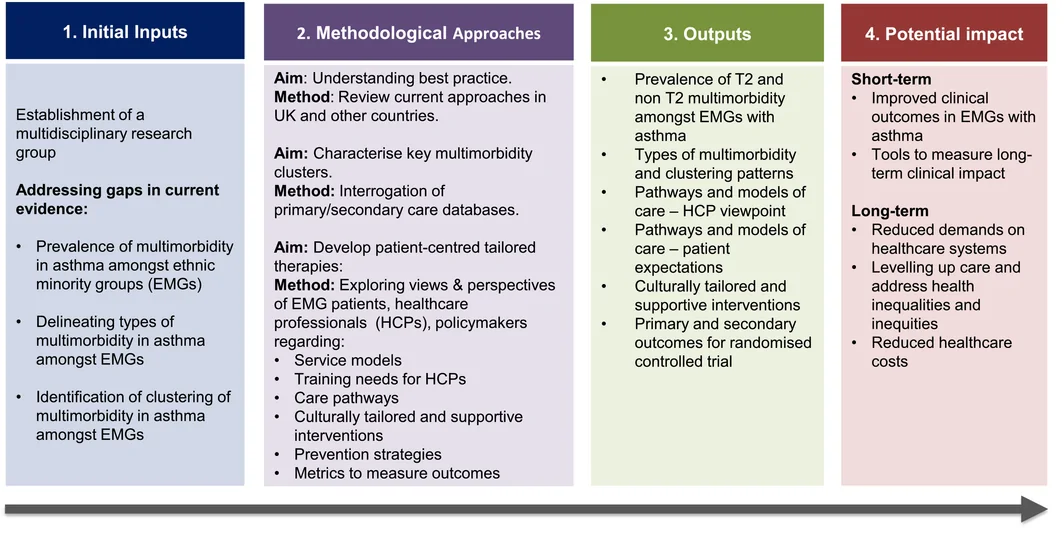
Logic model summarising strategic approach to research. Logic model addressing current gaps in knowledge via development of multistranded research programme.

**FIGURE 4:**
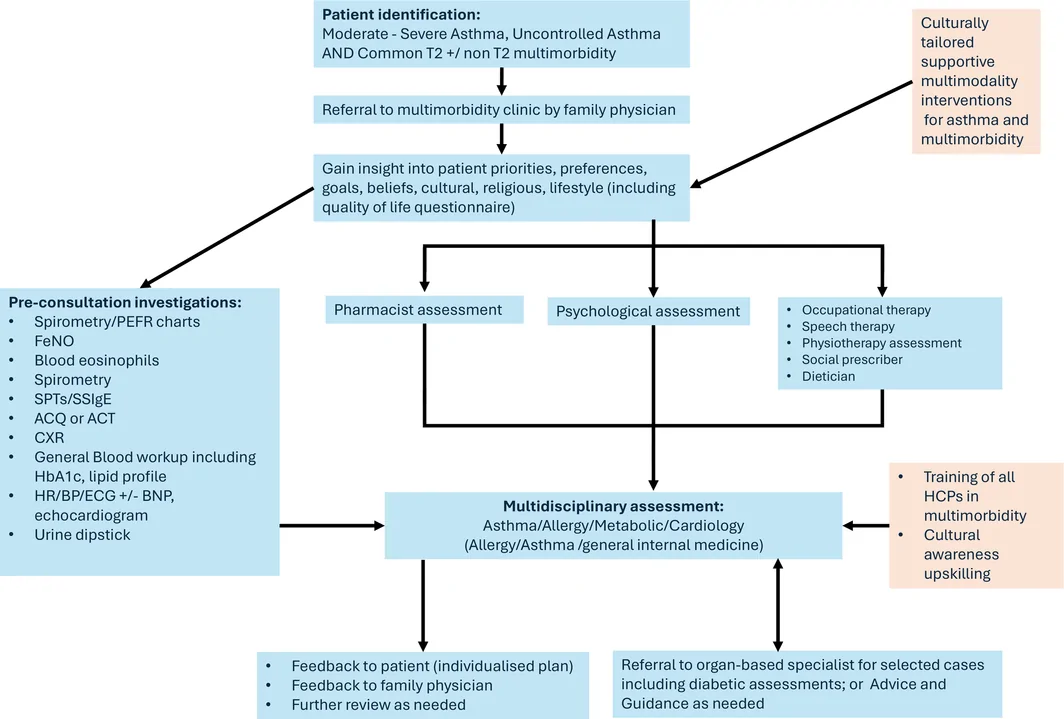
A multi‐dimensional service model for asthma with multimorbidity amongst ethnic minority groups. A multidimensional culturally tailored service model for asthma and multimorbidity amongst ethnic minority groups. ACQ, Asthma control questionnaire; ACT, Asthma control test; BNP, B‐type natriuretic peptide; BP, Blood pressure; CXR, Chest X ray; ECG, Electrocardiogram; FeNO, Fractional exhaled nitric oxide; HCP, Healthcare professional; HR, Heart rate; PEFR, Peak expiratory flow rate.

Figures 2, 3, 4 illustrate possible interventions for asthma with multimorbidity amongst EMGs including a logic model.

## 4 Gaps in Evidence in Asthma and Multimorbidity Amongst EMGs in High Income Countries

The selection of T2 and non‐T2 multimorbidity in all published asthma and multimorbidity studies thus far has been based on investigator judgement and experience rather than an evidence‐based hierarchical prevalence estimate data in the local population. A number of areas warrant further research. First, a population‐based study employing a joined‐up interrogation of primary care and secondary care databases to determine the prevalence of asthma with T2 and non T2 multimorbidity amongst EMGs, delineating hierarchical occurrence in different age groups supplemented with a cluster analysis to describe patterns of associations and link to socio‐demographic variables and clinical outcomes might feed into shaping risk stratification strategies. Second, gaining deeper insight into views and perspectives of EMG patients and their carers regarding their lived experiences of receiving care for asthma and multimorbidity as per current standard of care might be helpful alongside experiences of healthcare professionals and healthcare managers in primary and secondary care involved in the delivery of asthma management amongst EMGs. Third, it is prudent to develop culturally tailored resources for patients and carers highlighting the importance of a holistic approach to asthma and multimorbidity and raising awareness regarding treatable traits, primary and secondary prevention measures, optimising pharmacotherapy with an aim to reduce polypharmacy might feed into improving clinical outcomes. Table 1 summarises some ‘proof of concept’ studies making a case for a multidimensional approach to clinical management of asthma and multimorbidity amongst EMGs and highlights the need for further research.

**TABLE 1:** Key ‘proof of concept’ studies in multimorbidity making a case for a multidimensional approach to clinical management and further research.

Author (country)	Study design	Key findings	Analysis and limitation
Puri et al. (India) [83]	58,975 adults (≥ 45 years) from the Longitudinal Ageing Study in India, 2017–2018 16 noncommunicable diseases (NCDs) included: asthma, cancer, chronic bronchitis, chronic heart disease, chronic renal failure, chronic obstructive pulmonary disease, diabetes, gastrointestinal disorder, high cholesterol, hypertension, musculoskeletal disorder, neurological and psychiatric, skin disease, stroke, thyroid disease and urinary incontinence. Latent class analysis for common occurring patterns Logistic regression to investigate association between disease patterns with socioeconomic, demographic, lifestyle and anthropometric characteristics	Top 5 conditions: Hypertension (26.9%), Gastrointestinal tract disorders (18.4%), m usculoskeletal disorders (16.2%), diabetes (11.7%) and skin diseases (5.2%) Hypertension was most frequent amongst disease combinations identified 6 patterns identified: ‘relatively healthy’ hypertension’ gastrointestinal disorders–hypertension–musculoskeletal disorders musculoskeletal disorders–hypertension–asthma’, ‘metabolic disorders’ complex cardiometabolic disorders' Disease patterns were associated with socioeconomic, demographic, lifestyle and anthropometric	NCDs based on self‐reporting No causality was tested This study was not specifically designed to study asthma and multimorbidity
Chanoine et al. (France) [51]	Analysis was *N* = 17,458 women participating in the Asthma‐E3N study Nested case–control study on asthma within the E3N study (2011–2012) General features and respiratory health, comprehensive outpatient dispensed medication database from 2004 onwards Cluster analysis identified asthma groups sharing similar medication profiles and association with adverse asthma events	Network‐based analysis revealed more multi‐medications amongst women with asthma than those without 3 patterns/categories identified: Few multimorbidity‐related medications (43.5%) Predominantly allergic multimorbidity‐related medications (32.8%) Predominantly metabolic multimorbidity‐related medications (23.7%) Compared with women in category ‘a’, those belonging to ‘b’ & ‘c’ had an increased risk of adverse asthma events (uncontrolled asthma, acute asthma and asthma HRQoL)	50% of patients in category ‘c’ had mental health conditions Holistic and continuity of care needed, where both physical and mental health conditions are managed Highlights the need to move away from a ‘one size fits all’ single disease guideline‐based approach to asthma management to a person‐based multimorbidity approach This study involved mainly French White patients, so data is not generalisable, and specifically cannot be extrapolated to EMGs
Domínguez‐Ortega et al. (Spain) [52]	Prospective observational study Predictors of adverse events in patients who presented with ≥ 1 asthma adverse events in the previous year in the MEchanism of Genesis and Evolution of Asthma (MEGA) cohort *N* = 486; 23.7% mild, 35% moderate, 41.3% severe 15 co‐morbidities studied including food allergy, allergic rhinitis, chronic sinusitis and NSAID exacerbated respiratory disease	Comorbidity detected in 56.4% (66.6% in severe asthma) Significant associations with adverse events: Bronchiectasis, chronic rhinosinusitis with nasal polyps, atopy, psychiatric illnesses, hyperlipidaemia, and hypertension Sputum eosinophil counts and TS inflammatory pattern	Involved Spanish patients, so is not generalisable in other populations, and specifically cannot be extrapolated to EMGs Multimorbidity's included were based on physician selection rather than evidence based hierarchical prevalence estimates in asthma
Pattanaik et al. (India) [66]	WHO Study on Global AGEing and Adult Health (SAGE) Wave 2 (2014–2015) Part of a multi‐national study in Low‐ and Middle‐Income Countries Indian data analysed on participants ≥ 50 years (7118 [53% females] out of a total sample of 11,818) 24% belonged to most affluent quintile	Hypertension was most frequent and was most frequent common first diagnosis Top 8 chronic conditions: Hypertension (22%) Arthritis (19%) Stroke (2.36%) Angina (3.27%) Diabetes (9.44%) Chronic lung disease (2%) Asthma (5.11%) Depression (2.3%) Dyads (7.7%) Triads (2.37%)	Provides proof of concept regarding asthma as amongst top 8 chronic conditions amongst people ≥ 50 years Not generalisable to other age groups Does not provide insight regarding asthma and multimorbidity association
Park et al. (Korea) [67]	Participants ≥ 50 years (8370; mean age—62.5 years; 54% females) from the 6th Korean National Health and Nutrition Examination Survey (2013–2015) 10 chronic diseases included (hypertension, dyslipidaemia, stroke, osteoarthritis, tuberculosis, asthma, allergic rhinitis, depression, diabetes mellitus, and thyroid disease) Regression analysis to investigate associations between multimorbidity patterns, and sociodemographic factors and health‐related quality of life	Top 6 chronic conditions: Hypertension (36%) Dyslipidaemia (22%) Osteoarthritis (20%) Diabetes mellitus (14%) Allergic rhinitis (8%) 3 patterns identified: Relatively healthy group (60.4%); number of co‐occurring diseases—mean (95% CI) 0.6 (0.6–0.6) Cardiometabolic conditions' group (27.8%); number of co‐occurring diseases—mean (95% CI) 2.6 (2.6–2.7) Arthritis, asthma, allergic rhinitis, depression, and thyroid disease’ group (11.8%); number of co‐occurring diseases—mean (95% CI) 2.9 (2.8–3.0) Risk factors: Female sex and lower socioeconomic group for categories b & c Poor health outcomes: Categories b & c	Study was not specifically designed to investigate asthma and multimorbidity However, provides proof‐of‐concept regarding association of asthma with other common long‐term conditions amongst adults ≥ 50 years and intersection with poor deprivation and poor clinical outcomes
Scelo et al. (International severe asthma registry) [45]	Cross‐sectional study, 22 countries *N* = 11,821patients 30 co‐existing long‐term conditions studied: potentially T2‐related (69% at least 1 condition) potentially oral corticosteroid‐non‐T2 related multimorbidity (67% at least 1 condition) multimorbidity mimicking or aggravating asthma (55% at least 1 condition)	57% had ≥ 3 co‐morbidities Nasal/sinus related conditions associated with significantly greater asthma exacerbations Oral corticosteroids related conditions had higher odds of long‐term corticosteroid usage Mimicking/exacerbating conditions had higher odds of asthma exacerbations Greater number of co‐existing conditions—worse clinical outcomes 9/11 non‐T2 multimorbidity were associated with higher odds of long‐term oral corticosteroid usage	Provides ‘proof‐of‐concept’ regarding importance of recognition of asthmatics with associated T2 and non‐T2 long‐term conditions due to an adverse impact on clinical outcomes Data on ethnicity not provided/analysed Majority were High Income Countries from Europe and North America Highlights a need for a holistic personalised clinical care for severe asthma as opposed to a single guideline standardised care for severe asthma
Pate et al. (USA) [63]	National Health Interview Survey data (2021) *N* = 7343 children and adolescents (2–17 years) and 29,329 adults (≥ 18 years)	8% ≥ 2 years—asthma and 52.3%—allergy symptoms Asthma‐T2 conditions prevalence higher amongst adult females compared to males and higher amongst boys compared to girls Asthma‐T2 conditions higher in those from deprived background as per federal poverty level < 100 and non‐Hispanic Black people This study did not identify enhanced risk of asthma attacks amongst children with asthma and co‐existing T2 conditions Adults with asthma and T2 conditions had an enhanced risk of ≥ 1 asthma exacerbation in the preceding 12 months	Survey data subjected to patient recall Relatively small sample size to conduct sub‐group analysis Study conducted during the COVID‐19 pandemic, so may have had a potentially protective effect on asthma exacerbation due to lower infection rate and low levels of air pollution Provides evidence regarding disparities in asthma and T2 conditions based on age, sex, ethnicity and deprivation Women, deprivation and non‐Hispanic Black ethnicity more likely to report asthma with T2‐condition/s
Patel et al. (USA) [84]	Cross‐sectional interview data from the National Health Interview Survey *N* = 66,790; period; 2007–2012; Age 0 to 17 years; EMGs included 5% of children had 1 or more co‐existing health condition (multimorbidity) and 9 co‐existing conditions were investigated including congenital heart disease, autism, muscular dystrophy, sickle cell, hypertension, diabetes, other heart condition, arthritis and cerebral palsy	Patients with asthma were 1.6 times more likely to manage co‐existing long‐term conditions compared to those without asthma The cumulative number of co‐existing long‐term conditions were associated with adverse outcomes from asthma including acute attacks, school absenteeism and hospital admissions	Provides proof of concept regarding higher prevalence of multimorbidity in children and young adults with asthma Subgroup analysis on EMGs were not reported Selection bias regarding co‐existing long‐term conditions T2 conditions were not investigated Survey data subject to reporting and health seeking behaviour BMI data not available
Simms‐Willams et al. (UK) [6]	Cohort study using linked primary care (Clinical Practice Research Datalink Aurum) and secondary care (Hospital Episode Statistics Admitted Patient Care) data Included ≥ 5 years with asthma *N* = 90,989 children aged 5–11 years; *N* = 114,927 adolescents aged 12–17 years and *N* = 1179 410 adults ≥ 18 years; period: 2017–2019 Outcomes: Primary—asthma‐related hospital admission; secondary—asthma‐related intensive care unit admission Adjusted incident rate ratios determined	Risk for asthma‐related hospital admissions: younger age, female, deprivation, EMGs Adults: smoking, obesity, atopic conditions, gastro‐oesophageal reflux disease, anxiety and depression	Limited number of non‐T2 long‐term conditions were included Asthma may be misdiagnosed in primary care Potential misclassification of asthma‐related hospital admission resulting in overestimation Potential under diagnosis of anxiety and depression Interaction between risk factors were not studied Emergency room admissions were not included in the dataset which result in underestimation of adverse clinical outcomes
Lin et al. (UK) [85]	Prospective cohort study (2006–2010) based on the UK Biobank linked to primary care records, hospital admission and death register data *N* = 51,335 with asthma and *N* = 395,890 without asthma Median follow up: 13.8 years Cox proportional hazards models and multi‐state models Followed up development of cardio‐metabolic disorders (CMD; type 2 diabetes, coronary heart disease, and stroke) and cardio‐metabolic multimorbidity (CMM)	60,033 participants (13.4%) developed CMD, of whom 7048 (1.6%) progressed to CMM Asthma was associated with enhanced risk of all the 3 CMDs and CMM (hazard ratio = 1.54, 95% CI 1.44–1.64) Effect greater in women	This study serves as a proof of concept for ‘holistic care’ in the context of asthma and multimorbidity Highlights potential benefit for strategies for early diagnosis, primary and secondary prevention of CMDs including addressing treatable traits relevant to CMDs
Verest et al. (The Netherlands) [86]	Healthy Life in an Urban Setting study (HELIUS); 2011–2015 Random sampling and questionnaire based *N* = 23,942; Dutch (*N* = 4582), South‐Asian Surinamese (*N* = 3258), African Surinamese (*N* = 4267), Ghanaian (*N* = 2282), Turkish (*N* = 3879) and Moroccan (*N* = 4094) Aged 18–70 years List of 20 conditions included as reported by participants in the preceding 12 months	After adjustment of socioeconomic status, adjusted odds ratio for multimorbidity significantly higher amongst EMGs Higher prevalence of multimorbidity amongst both men and women from EMGs Earlier onset multimorbidity by 1–3 decades amongst EMGs	Asthma and multimorbidity not specifically studied Data showed an enhanced risk of multimorbidity amongst Dutch EMGs Data limited by self‐reporting Higher age limit set at 70 years, so not inclusive of the elderly who are likely to have a greater burden of multimorbidity
George et al. (USA) [87]	*N* = 402,403; ≥ 12 years; moderate‐to‐severe asthma Data extracted from electronic health records 2012–2018; risk factors for uncontrolled asthma determined as per Cox proportional hazard model Ethnicity: African‐American 13.5%; Asian 1.5%; White 78.9%; Other/unknown 6.1% Good geographical cover of USA including Midwest 53.2%, Northeast 12%, South 23.9%, West 7.3%, Other/unknown 3.6%	Hazard ratios: African American—2.08 Hispanic—1.34 Medicaid insurance—1.71 Age 12 to < 18 years—1.20 BMI ≥ 35 kg/m^2^–1.20 Female sex—1.19 Blood eosinophils ≥ 300/μL—1.40 Food allergy—1.31 Pneumonia—1.3	Asthma severity defined as per medication use in the last 12 months Data provides evidence of intersection between poor clinical outcome and ethnicity, co‐existing long‐term condition/s as well as other co‐variates Socio‐economic status was not included in analysis > 90% participants were Caucasian White; sub‐analysis for EMGs not presented
Arias et al. (USA) [88]	Secondary analysis (2008–2013) of National Survey on Drug Use and Health *N* = 206,993 adults Investigated association between asthma and mental health (past year serious psychological distress [SPD] and doctor diagnosis of depression) Bivariate Chi‐square and multivariable regression	Current and lifetime asthma was significantly associated with past year SPD and depression amongst non‐Hispanic White, Hispanics and African‐American people	Cross‐sectional study and does not provide evidence for causality Asians not included in this study Self‐reported data has limitations and subject to underestimates

## 5 Conclusion

Asthma places a significant burden at both individual and societal levels. It frequently occurs alongside multiple long‐term health conditions within a framework of multimorbidity. Emerging evidence shows that greater multimorbidity burden is associated with worse asthma outcomes whilst addressing treatable traits and co‐existing T2 and non‐T2 long‐term conditions can improve asthma outcomes. People from EMGs often from a deprived socioeconomic background, experience an increased health disadvantage and detrimental impacts from multimorbidity There is now a clear imperative need to better understand the nature and impacts of asthma with multimorbidity in EMGs and use that new knowledge to design bespoke and holistic clinical care pathways and address health inequalities and improve the health status, and long‐term prognosis. This requires a multipronged joined up and an integrated approach involving patients, patient organisations, policy makers and healthcare providers. This includes the development of novel culturally tailored multidisciplinary approaches to support the bespoke needs of EMGs with asthma, improve access to healthcare for marginalised and deprived hard to reach people in the community, strategies to improve health literacy, upskill healthcare professionals in cultural awareness and facilitate diverse leadership at all levels in health services with an aim to drive new and equitable initiatives to eliminate institutional racism where it exists.

## Author Contributions

All authors contributed to conceptualisation. M.T.K. led the write up in collaboration with S.M., B.A., A.F., R.K.n., R.K.u.r., and A.H.M. All authors edited and approved the final version of the manuscript.

## Conflicts of Interest

M.T.K. received research grants from NIHR, FSA, MRC CiC and GCRF. He is clinical lead for Equality, Diversity and Inclusion for College of Medicine and Health, University of Birmingham. A.H.M. declares no conflicts of interest in relation to this work. He otherwise received personal and institutional payments for talks, advisory board meetings, sponsorships to attend conferences from AZ, GSK, Sanofi and Chiesi, and received a research grant from NIHR. Other co‐authors have no conflicts of interest to declare.

## Data Availability

The authors have nothing to report.

## References

[1] Global Asthma Network , “The Global Asthma Report 2018,” (2018), https://www.globalasthmareport.org/resources/global_asthma_report_2018.pdf.

[2] Asthma UK , “On the Edge: How Inequality Affects People With Asthma,” (2018).

[3] Asthma UK , “Hospitalisation Dashboard,” https://wwwukasthmadashboardcouk/united‐kingdom.

[4] M. Morris , L. M. Woods , and B. Rachet , “A Novel Ecological Methodology for Constructing Ethnic‐Majority Life Tables in the Absence of Individual Ethnicity Information,” Journal of Epidemiology and Community Health 69, no. 4 (2015): 361–367. 25563743 10.1136/jech-2014-204210 PMC4392229

[5] A. P. Baptist , W. Zhou , B. E. Chipps , D. D. Carstens , and C. S. Ambrose , “Socioeconomic Disparities Explain Increased Exacerbations Among Black Patients With Severe Asthma,” Journal of Allergy and Clinical Immunology. In Practice 13, no. 12 (2025): 3277–3285. 40645374 10.1016/j.jaip.2025.06.037

[6] N. Simms‐Williams , P. Nagakumar , R. Thayakaran , et al., “Risk Factors for Asthma‐Related Hospital and Intensive Care Admissions in Children, Adolescents and Adults: A Cohort Study Using Primary and Secondary Care Data,” BMJ Open Respiratory Research 11, no. 1 (2024): e001746. 10.1136/bmjresp-2023-001746 PMC11086188 38692709

[7] GOV.UK , “People Living in Deprived Neighbourhoods,” https://www.ethnicity‐facts‐figures.service.gov.uk/uk‐population‐by‐ethnicity/demographics/people‐living‐in‐deprived‐neighbourhoods/latest.

[8] A. Subramanian , N. J. Adderley , G. V. Gkoutos , K. M. Gokhale , K. Nirantharakumar , and M. T. Krishna , “Ethnicity‐Based Differences in the Incident Risk of Allergic Diseases and Autoimmune Disorders: A UK‐Based Retrospective Cohort Study of 4.4 Million Participants,” Clinical and Experimental Allergy 51, no. 1 (2021): 144–147. 32946613 10.1111/cea.13741

[9] E. S. Petherick , N. Pearce , J. Sunyer , and J. Wright , “Ethnic and Socio‐Economic Differences in the Prevalence of Wheeze, Severe Wheeze, Asthma, Eczema and Medication Usage at 4 Years of Age: Findings From the Born in Bradford Birth Cohort,” Respiratory Medicine 119 (2016): 122–129. 27692132 10.1016/j.rmed.2016.08.017

[10] G. Netuveli , B. Hurwitz , M. Levy , et al., “Ethnic Variations in UK Asthma Frequency, Morbidity, and Health‐Service Use: A Systematic Review and Meta‐Analysis,” Lancet 365, no. 9456 (2005): 312–317. 15664226 10.1016/S0140-6736(05)17785-X

[11] Centers for Disease Control and Prevention , Most Recent National Asthma Data (U.S. Department of Health and Human Services, 2019).

[12] Y. Wang , K. J. Allen , N. H. A. Suaini , R. L. Peters , A. L. Ponsonby , and J. J. Koplin , “Asian Children Living in Australia Have a Different Profile of Allergy and Anaphylaxis Than Australian‐Born Children: A State‐Wide Survey,” Clinical and Experimental Allergy 48, no. 10 (2018): 1317–1324. 30025179 10.1111/cea.13235

[13] C. M. Davis , A. J. Apter , A. Casillas , et al., “Health Disparities in Allergic and Immunologic Conditions in Racial and Ethnic Underserved Populations: A Work Group Report of the AAAAI Committee on the Underserved,” Journal of Allergy and Clinical Immunology 147, no. 5 (2021): 1579–1593. 33713767 10.1016/j.jaci.2021.02.034

[14] M. T. Krishna , S. Hackett , C. Bethune , and A. T. Fox , “Achieving Equitable Management of Allergic Disorders and Primary Immunodeficiency in a Black, Asian and Minority Ethnic Population,” Clinical and Experimental Allergy 50, no. 8 (2020): 880–883. 32936482 10.1111/cea.13698

[15] C. J. Jones , P. Paudyal , R. M. West , et al., “Burden of Allergic Disease Among Ethnic Minority Groups in High‐Income Countries,” Clinical and Experimental Allergy 52, no. 5 (2022): 604–615. 35306712 10.1111/cea.14131 PMC9324921

[16] S. B. Sarpong , R. G. Hamilton , P. A. Eggleston , and N. F. Adkinson, Jr. , “Socioeconomic Status and Race as Risk Factors for Cockroach Allergen Exposure and Sensitization in Children With Asthma,” Journal of Allergy and Clinical Immunology 97, no. 6 (1996): 1393–1401. 8648037 10.1016/s0091-6749(96)70209-9

[17] A. H. Mansur , J. Marsh , A. Bahron , et al., “Difficult‐To‐Treat Asthma Patients From Ethnic Minority Groups in Central England Are at an Enhanced Risk of House Dust Mite Sensitisation,” Clinical and Translational Allergy 13, no. 10 (2023): e12303. 37876034 10.1002/clt2.12303 PMC10560749

[18] A. Sheikh , M. F. Steiner , G. Cezard , et al., “Ethnic Variations in Asthma Hospital Admission, Readmission and Death: A Retrospective, National Cohort Study of 4.62 Million People in Scotland,” BMC Medicine 14 (2016): 3. 26755184 10.1186/s12916-015-0546-6 PMC4710027

[19] S. Moitra , A. Adan , M. Akgun , et al., “Less Social Deprivation Is Associated With Better Health‐Related Quality of Life in Asthma and Is Mediated by Less Anxiety and Better Sleep Quality,” Journal of Allergy and Clinical Immunology. In Practice 11, no. 7 (2023): 2115–2124. 37087095 10.1016/j.jaip.2023.03.052

[20] T. Osborne , G. Walters , R. Baretto , and M. T. Krishna , “Disparities in Food Allergy Amongst British South Asian Adult Patients in Central England,” World Allergy Organization Journal 18, no. 9 (2025): 101099. 40977758 10.1016/j.waojou.2025.101099 PMC12446536

[21] R. J. Buka , R. J. Crossman , C. L. Melchior , et al., “Anaphylaxis and Ethnicity: Higher Incidence in British South Asians,” Allergy 70, no. 12 (2015): 1580–1587. 26214068 10.1111/all.12702

[22] F. E. Simons , L. R. Ardusso , M. B. Bilo , et al., “World Allergy Organization Anaphylaxis Guidelines: Summary,” Journal of Allergy and Clinical Immunology 127, no. 3 (2011): 587–593.e1‐22. 21377030 10.1016/j.jaci.2011.01.038

[23] R. S. Pumphrey , “Lessons for Management of Anaphylaxis From a Study of Fatal Reactions,” Clinical and Experimental Allergy 30, no. 8 (2000): 1144–1150. 10931122 10.1046/j.1365-2222.2000.00864.x

[24] R. S. Pumphrey , “Fatal Anaphylaxis in the UK, 1992–2001,” Novartis Foundation Symposium 257 (2004): 116–128. 15025395

[25] E. Jerschow , R. Y. Lin , M. M. Scaperotti , and A. P. McGinn , “Fatal Anaphylaxis in the United States, 1999–2010: Temporal Patterns and Demographic Associations,” Journal of Allergy and Clinical Immunology 134, no. 6 (2014): 1318–1328.e7. 25280385 10.1016/j.jaci.2014.08.018 PMC4260987

[26] S. Nazario , L. Gonzalez‐Sepulveda , B. Telon‐Sosa , and E. L. Suarez‐Perez , “Inequalities in Asthma Mortality by Ethnicity and Race in the United States and Puerto Rico,” Journal of Allergy and Clinical Immunology. In Practice 10, no. 8 (2022): 2178–2180. 35662525 10.1016/j.jaip.2022.04.023 PMC10329183

[27] A. Gawlik‐Lipinski , S. Hassen , M. Ramphul , et al., “Risk Factors for Life‐Threatening Asthma Attacks and Asthma‐Related Mortality in Children‐A Systematic Review,” Pediatric Pulmonology 60, no. 8 (2025): e71255. 40827729 10.1002/ppul.71255 PMC12391745

[28] CDC , “CDC Data 2001–21,” https://www.cdc.gov/asthma/asthma_stats/asthma‐deaths_2001‐2021.html.

[29] C. Farmer , E. Fenu , N. O'Flynn , and B. Guthrie , “Clinical Assessment and Management of Multimorbidity: Summary of NICE Guidance,” BMJ 354 (2016): i4843. 27655884 10.1136/bmj.i4843

[30] K. Khunti , F. S. Mair , P. Dorairaj , and J. Valabhji , “Healthcare Solutions for People With Multiple Long Term Conditions,” BMJ 391 (2025): r2563. 41365604 10.1136/bmj.r2563

[31] J. Valabhji , E. Barron , A. Pratt , et al., “Prevalence of Multiple Long‐Term Conditions (Multimorbidity) in England: A Whole Population Study of Over 60 Million People,” Journal of the Royal Society of Medicine 117, no. 3 (2024): 104–117. 37905525 10.1177/01410768231206033 PMC11046366

[32] A. K. Kavitha , J. S. Kshatri , M. van den Akker , M. A. Hussain , H. Bhattacharya , and S. Pati , “Epidemiology of Multimorbidity Among Indigenous Adults: Insights From a Large‐Scale Population Survey of 53 Different Indigenous Groups in East India,” Glob Epidemiol 10 (2025): 100226. 41158847 10.1016/j.gloepi.2025.100226 PMC12556326

[33] A. Sinha , S. Kerketta , S. Ghosal , S. Kanungo , and S. Pati , “Multimorbidity Among Urban Poor in India: Findings From LASI, Wave‐1,” Frontiers in Public Health 10 (2022): 881967. 35719649 10.3389/fpubh.2022.881967 PMC9201724

[34] T. R. Flores , A. Rodrigues , R. G. Neves , et al., “The Risk of Multimorbidity Associated With Overweight and Obesity: Data From the Brazilian National Health Survey 2013,” Journal of Obesity & Metabolic Syndrome 30, no. 2 (2021): 155–162. 33972471 10.7570/jomes20110 PMC8277594

[35] S. Pati , P. Mahapatra , R. Dwivedi , et al., “Multimorbidity and Its Outcomes Among Patients Attending Psychiatric Care Settings: An Observational Study From Odisha, India,” Frontiers in Public Health 8 (2020): 616480. 33968863 10.3389/fpubh.2020.616480 PMC8096979

[36] N. Goel , I. Biswas , and K. Chattopadhyay , “Risk Factors of Multimorbidity Among Older Adults in India: A Systematic Review and Meta‐Analysis,” Health Science Reports 7, no. 2 (2024): e1915. 38420204 10.1002/hsr2.1915 PMC10900089

[37] GBD , “2015 Mortality and Causes of Death Collaborators. Global, Regional, and National Life Expectancy, All‐Cause Mortality, and Cause‐Specific Mortality for 249 Causes of Death, 1980–2015: A Systematic Analysis for the Global Burden of Disease Study 2015,” Lancet 388 (2016): 1499–1544. 10.1016/S0140-6736(16)31012-1 PMC5388903 27733281

[38] S. E. Vollset , H. Ababneh , Y. Abate , et al., “Burden of Disease Scenarios for 204 Countries and Territories, 2022–2050: A Forecasting Analysis for the Global Burden of Disease Study,” Lancet 403, no. 10440 (2021): 2204–2256. 10.1016/S0140-6736(24)00685-8 PMC11121021 38762325

[39] C. Buffel du Vaure , A. Dechartres , C. Battin , P. Ravaud , and I. Boutron , “Exclusion of Patients With Concomitant Chronic Conditions in Ongoing Randomised Controlled Trials Targeting 10 Common Chronic Conditions and Registered at ClinicalTrials.Gov: A Systematic Review of Registration Details,” BMJ Open 6, no. 9 (2016): e012265. 10.1136/bmjopen-2016-012265 PMC5051474 27678540

[40] K. Jindai , C. M. Nielson , B. A. Vorderstrasse , and A. R. Quinones , “Multimorbidity and Functional Limitations Among Adults 65 or Older, NHANES 2005–2012,” Preventing Chronic Disease 13 (2016): E151. 27809419 10.5888/pcd13.160174 PMC5094859

[41] J. Pearson‐Stuttard , M. Ezzati , and E. W. Gregg , “Multimorbidity‐A Defining Challenge for Health Systems,” Lancet Public Health 4, no. 12 (2019): e599–e600. 31812234 10.1016/S2468-2667(19)30222-1

[42] A. Head , K. Fleming , C. Kypridemos , P. Schofield , J. Pearson‐Stuttard , and M. O'Flaherty , “Inequalities in Incident and Prevalent Multimorbidity in England, 2004–19: A Population‐Based, Descriptive Study,” Lancet Healthy Longevity 2, no. 8 (2021): e489–e497. 36097998 10.1016/S2666-7568(21)00146-X

[43] C. Mbuya‐Bienge , M. Simard , M. Gaulin , B. Candas , and C. Sirois , “Does Socio‐Economic Status Influence the Effect of Multimorbidity on the Frequent Use of Ambulatory Care Services in a Universal Healthcare System? A Population‐Based Cohort Study,” BMC Health Services Research 21, no. 1 (2021): 202. 33676497 10.1186/s12913-021-06194-w PMC7937264

[44] F. Eto , M. Samuel , R. Henkin , et al., “Ethnic Differences in Early Onset Multimorbidity and Associations With Health Service Use, Long‐Term Prescribing, Years of Life Lost, and Mortality: A Cross‐Sectional Study Using Clustering in the UK Clinical Practice Research Datalink,” PLoS Medicine 20, no. 10 (2023): e1004300. 37889900 10.1371/journal.pmed.1004300 PMC10610074

[45] G. Scelo , C. A. Torres‐Duque , J. Maspero , et al., “Analysis of Comorbidities and Multimorbidity in Adult Patients in the International Severe Asthma Registry,” Annals of Allergy, Asthma & Immunology 132, no. 1 (2024): 42–53. 10.1016/j.anai.2023.08.021 37640263

[46] R. J. Kurukulaaratchy , A. Freeman , A. T. Bansal , et al., “Evaluation of the Effect of Multimorbidity on Difficult‐To‐Treat Asthma Using a Novel Score (MiDAS): A Multinational Study of Asthma Cohorts,” Lancet Respiratory Medicine 13, no. 9 (2025): 821–832. 40645203 10.1016/S2213-2600(25)00135-3

[47] L. H. M. Lim , Y. R. Juang , S. H. Chotirmall , et al., “Economic Burden of Asthma Multimorbidity in Singapore: Shadow Costs of Steroid Use,” World Allergy Organization Journal 18, no. 12 (2025): 101146. 41394324 10.1016/j.waojou.2025.101146 PMC12701672

[48] T. R. Tay , P. H. Pang , M. F. Liew , et al., “Ethnic Variation in South East Asian Patients With Severe Asthma: A First Look at the Singapore Severe Asthma Registry (SSAR) and Implications on Clinical Practice,” Respiratory Medicine 245 (2025): 108199. 40482918 10.1016/j.rmed.2025.108199

[49] A. R. Mulick , A. D. Henderson , D. Prieto‐Merino , et al., “Novel Multimorbidity Clusters in People With Eczema and Asthma: A Population‐Based Cluster Analysis,” Scientific Reports 12, no. 1 (2022): 21866. 36529816 10.1038/s41598-022-26357-x PMC9760185

[50] P. E. Pfeffer , H. Rupani , and A. De Simoni , “Bringing the Treatable Traits Approach to Primary Care Asthma Management,” Frontiers in Allergy 4 (2023): 1240375. 37799134 10.3389/falgy.2023.1240375 PMC10548136

[51] S. Chanoine , M. Sanchez , I. Pin , et al., “Multimorbidity Medications and Poor Asthma Prognosis,” European Respiratory Journal 51, no. 4 (2018): 1702114. 29545275 10.1183/13993003.02114-2017

[52] J. Dominguez‐Ortega , J. A. Luna‐Porta , J. M. Olaguibel , et al., “Exacerbations Among Patients With Asthma Are Largely Dependent on the Presence of Multimorbidity,” Journal of Investigational Allergology & Clinical Immunology 33, no. 4 (2023): 281–288. 35503227 10.18176/jiaci.0816

[53] W. H. Cheng , K. Borsos , Z. Wang , et al., “Burden of Disease and Unmet Clinical Needs in Pediatric Patients With Asthma: Real‐World Experience From the United States and Five European Countries,” Journal of Asthma 62, no. 10 (2025): 1698–1711. 10.1080/02770903.2025.2513059 40488436

[54] A. Freeman , S. Rink , T. Aruna , et al., “Multimorbidity Phenotypes and Associated Characteristics in Severe Asthma: An Observational Study of European Severe Asthma Registries,” Lancet Regional Health‐Europe 63 (2026): 101600. 41694692 10.1016/j.lanepe.2026.101600 PMC12906202

[55] A. Shackleford , L. G. Heaney , C. Redmond , P. J. McDowell , and J. Busby , “Clinical Remission Attainment, Definitions, and Correlates Among Patients With Severe Asthma Treated With Biologics: A Systematic Review and Meta‐Analysis,” Lancet Respiratory Medicine 13, no. 1 (2025): 23–34. 39549709 10.1016/S2213-2600(24)00293-5

[56] B. Hayanga , M. Stafford , and L. Becares , “Ethnic Inequalities in Multiple Long‐Term Health Conditions in the United Kingdom: A Systematic Review and Narrative Synthesis,” BMC Public Health 23, no. 1 (2023): 178. 36703163 10.1186/s12889-022-14940-w PMC9879746

[57] C. Razieh , F. Zaccardi , J. Miksza , et al., “Differences in the Risk of Cardiovascular Disease Across Ethnic Groups: UK Biobank Observational Study,” Nutrition, Metabolism, and Cardiovascular Diseases 32, no. 11 (2022): 2594–2602. 10.1016/j.numecd.2022.08.002 36064688

[58] H. M. Gonzalez , W. Tarraf , K. E. Whitfield , and W. A. Vega , “The Epidemiology of Major Depression and Ethnicity in the United States,” Journal of Psychiatric Research 44, no. 15 (2010): 1043–1051. 20537350 10.1016/j.jpsychires.2010.03.017 PMC2963677

[59] P. Irizar , H. Taylor , D. Kapadia , et al., “The Prevalence of Common Mental Disorders Across 18 Ethnic Groups in Britain During the COVID‐19 Pandemic: Evidence for Equality National Survey (EVENS),” Journal of Affective Disorders 358 (2024): 42–51. 38705522 10.1016/j.jad.2024.05.026

[60] F. Rees , M. Doherty , M. Grainge , G. Davenport , P. Lanyon , and W. Zhang , “The Incidence and Prevalence of Systemic Lupus Erythematosus in the UK, 1999‐2012,” Annals of the Rheumatic Diseases 75, no. 1 (2016): 136–141. 25265938 10.1136/annrheumdis-2014-206334 PMC4717400

[61] S. Sutaria , R. Mathur , and S. A. Hull , “Does the Ethnic Density Effect Extend to Obesity? A Cross‐Sectional Study of 415 166 Adults in East London,” BMJ Open 9, no. 5 (2019): e024779. 10.1136/bmjopen-2018-024779 PMC6549644 31154296

[62] W. Gibson‐Scipio , V. Dinaj , A. Hall , A. Kormelink , J. M. Bruzzese , and K. K. MacDonell , “Anxiety, Depression and Global Distress Among African American Young Adults With Uncontrolled Asthma,” Journal of Asthma 60, no. 10 (2023): 1836–1842. 10.1080/02770903.2023.2193632 PMC10524604 36952598

[63] C. A. Pate , L. J. Akinbami , C. Johnson , J. Hsu , and H. S. Zahran , “Asthma and Allergy Comorbidity Among the US Population Aged 2 Years or Older, National Health Interview Survey, 2021,” Public Health Reports 140, no. 5–6 (2025): 540–550. 40842192 10.1177/00333549251358658 PMC12373655

[64] A. Bisquera , E. B. Turner , L. Ledwaba‐Chapman , et al., “Inequalities in Developing Multimorbidity Over Time: A Population‐Based Cohort Study From an Urban, Multi‐Ethnic Borough in the United Kingdom,” Lancet Regional Health Europen 12 (2022): 100247. 10.1016/j.lanepe.2021.100247 PMC8640725 34901910

[65] M. Engelkes , M. A. de Ridder , E. Svensson , et al., “Multinational Cohort Study of Mortality in Patients With Asthma and Severe Asthma,” Respiratory Medicine 165 (2020): 105919. 32174450 10.1016/j.rmed.2020.105919

[66] A. A. Pattanaik , R. U. A. Hashmi , R. S. Dandsena , et al., “Multimorbidity in India: An Analysis of Prevalence and Patterns of Chronicity of Diseases Among Older Adults Using Study on Global AGEing and Adult Health (SAGE) Wave 2,” BMJ Public Health 3, no. 2 (2025): e002143. 41069972 10.1136/bmjph-2024-002143 PMC12506212

[67] B. Park , H. A. Lee , and H. Park , “Use of Latent Class Analysis to Identify Multimorbidity Patterns and Associated Factors in Korean Adults Aged 50 Years and Older,” PLoS One 14, no. 11 (2019): e0216259. 31721778 10.1371/journal.pone.0216259 PMC6853322

[68] Z. Gassase , I. Sinha , G. A. Davies , et al., “Inequalities in Asthma Outcomes in England: A National Cohort Study,” European Respiratory Journal 66 (2025): PA2622.

[69] M. R. Janevic , K. R. Ellis , G. M. Sanders , B. W. Nelson , and N. M. Clark , “Self‐Management of Multiple Chronic Conditions Among African American Women With Asthma: A Qualitative Study,” Journal of Asthma 51, no. 3 (2014): 243–252. 10.3109/02770903.2013.860166 PMC4234073 24161047

[70] A. Agusti , E. Bel , M. Thomas , et al., “Treatable Traits: Toward Precision Medicine of Chronic Airway Diseases,” European Respiratory Journal 47, no. 2 (2016): 410–419. 26828055 10.1183/13993003.01359-2015

[71] V. M. McDonald , V. L. Clark , L. Cordova‐Rivera , P. A. B. Wark , K. J. Baines , and P. G. Gibson , “Targeting Treatable Traits in Severe Asthma: A Randomised Controlled Trial,” European Respiratory Journal 55, no. 3 (2020): 1901509. 31806719 10.1183/13993003.01509-2019

[72] T. Lin , J. Pham , E. Denton , et al., “Trait Profiles in Difficult‐To‐Treat Asthma: Clinical Impact and Response to Systematic Assessment,” Allergy 78, no. 9 (2023): 2418–2427. 36940306 10.1111/all.15719

[73] E. Denton , J. Lee , T. Tay , et al., “Systematic Assessment for Difficult and Severe Asthma Improves Outcomes and Halves Oral Corticosteroid Burden Independent of Monoclonal Biologic Use,” Journal of Allergy and Clinical Immunology. In Practice 8, no. 5 (2020): 1616–1624. 31954193 10.1016/j.jaip.2019.12.037

[74] B. Ainsworth , A. Patel , C. Eyles , G. E. Davies , and M. Thomas , “Feasibility and Acceptability of a Group Mindfulness Intervention in a Difficult Asthma Clinic,” Mindfulness 11, no. 7 (2020): 1734–1746.

[75] Y. C. Huang and A. A. Garcia , “Culturally‐Tailored Interventions for Chronic Disease Self‐Management Among Chinese Americans: A Systematic Review,” Ethnicity & Health 25, no. 3 (2020): 465–484. 29385815 10.1080/13557858.2018.1432752

[76] S. Ahmed , L. Steed , K. Harris , S. J. C. Taylor , and H. Pinnock , “Interventions to Enhance the Adoption of Asthma Self‐Management Behaviour in the South Asian and African American Population: A Systematic Review,” NPJ Primary Care Respiratory Medicine 28, no. 1 (2018): 5. 10.1038/s41533-017-0070-6 PMC5814446 29449558

[77] D. Vincent , M. M. McEwen , J. T. Hepworth , and C. S. Stump , “The Effects of a Community‐Based, Culturally Tailored Diabetes Prevention Intervention for High‐Risk Adults of Mexican Descent,” Diabetes Educator 40, no. 2 (2014): 202–213. 24510942 10.1177/0145721714521020 PMC6383713

[78] I. C. Williams , S. W. Utz , I. Hinton , G. Yan , R. Jones , and K. Reid , “Enhancing Diabetes Self‐Care Among Rural African Americans With Diabetes: Results of a Two‐Year Culturally Tailored Intervention,” Diabetes Educator 40, no. 2 (2014): 231–239. 24478047 10.1177/0145721713520570 PMC4692724

[79] C. Salisbury , M. S. Man , P. Bower , et al., “Management of Multimorbidity Using a Patient‐Centred Care Model: A Pragmatic Cluster‐Randomised Trial of the 3D Approach,” Lancet 392, no. 10141 (2018): 41–50. 29961638 10.1016/S0140-6736(18)31308-4 PMC6041724

[80] D. M. Berwick , T. W. Nolan , and J. Whittington , “The Triple Aim: Care, Health, and Cost,” Health Affairs (Millwood) 27, no. 3 (2008): 759–769. 10.1377/hlthaff.27.3.759 18474969

[81] B. Ainsworth , E. Chatburn , A. T. Bansal , et al., “What Bothers Severe Asthma Patients Most? A Paired Patient‐Clinician Study Across Seven European Countries,” ERJ Open Research 9, no. 3 (2023): 2022. 10.1183/23120541.00717-2022 PMC10227631 37260457

[82] C. Bell , P. Vedsted , D. G. A. Kraus , et al., “Clinic for Multimorbidity: An Innovative Approach to Integrate General Practice and Specialized Health Care Services,” International Journal of Integrated Care 23, no. 2 (2023): 25. 37333774 10.5334/ijic.7015 PMC10275160

[83] P. Puri , S. K. Singh , and S. Pati , “Identifying Non‐Communicable Disease Multimorbidity Patterns and Associated Factors: A Latent Class Analysis Approach,” BMJ Open 12, no. 7 (2022): e053981. 10.1136/bmjopen-2021-053981 PMC9277367 35820748

[84] M. R. Patel , H. L. Leo , A. P. Baptist , Y. Cao , and R. W. Brown , “Asthma Outcomes in Children and Adolescents With Multiple Morbidities: Findings From the National Health Interview Survey,” Journal of Allergy and Clinical Immunology 135, no. 6 (2015): 1444–1449. 25533524 10.1016/j.jaci.2014.11.008 PMC13749053

[85] J. Lin , Y. Cheng , Y. Zhang , et al., “Associations of Asthma With Cardiometabolic Diseases and Multimorbidity: A Cohort Study in the UK Biobank,” NPJ Primary Care Respiratory Medicine 36, no. 1 (2025): 10. 10.1038/s41533-025-00474-2 PMC12864751 41461656

[86] W. Verest , H. Galenkamp , B. Spek , M. B. Snijder , K. Stronks , and I. G. M. van Valkengoed , “Do Ethnic Inequalities in Multimorbidity Reflect Ethnic Differences in Socioeconomic Status? The HELIUS Study,” European Journal of Public Health 29, no. 4 (2019): 687–693. 30768174 10.1093/eurpub/ckz012 PMC6660190

[87] M. George , C. A. Camargo, Jr. , A. Burnette , et al., “Racial and Ethnic Minorities at the Highest Risk of Uncontrolled Moderate‐To‐Severe Asthma: A United States Electronic Health Record Analysis,” Journal of Asthma Allergy 16 (2023): 567–577. 37200709 10.2147/JAA.S383817 PMC10187653

[88] D. Arias , B. J. Becerra , and M. B. Becerra , “Racial and Ethnic Differences in Asthma and Mental Health Among US Adults: Results From the National Survey on Drug Use and Health,” Journal of Asthma 52, no. 7 (2015): 715–720. 10.3109/02770903.2015.1005843 25584661

